# Drying methods affect physicochemical and functional characteristics of *Clanis Bilineata Tingtauica Mell* protein

**DOI:** 10.3389/fnut.2022.1053422

**Published:** 2022-11-09

**Authors:** Shuya Wang, Liqiong Niu, Bin Zhou, Yao Peng, Xinquan Yang, Yingbin Shen, Shugang Li

**Affiliations:** ^1^School of Life Sciences, Guangzhou University, Guangzhou, China; ^2^School of Food and Biological Engineering, Hubei University of Technology, Wuhan, China; ^3^Engineering Research Center of Bio-process, Ministry of Education, School of Food and Biological Engineering, Hefei University of Technology, Hefei, China

**Keywords:** *Clanis Bilineata Tingtauica Mell* protein, drying methods, structure, functional properties, digestive properties

## Abstract

Clanis Bilineata Tingtauica Mell Protein (CBTMP) was a kind of natural full-price protein which has a bright application prospect in the food industry. Since the functional properties of protein can be significantly affected by drying method, this study aims to explore the effect of different drying methods, namely freeze drying (FD), vacuum drying (VD),and hot-air drying (HD) on the structure and functional properties of CBTMP. The results showed that the degree of oxidation of CBTMP was found to be in the following order: HD > VD > FD. Functional characteristics revealed that the CBTMP prepared by VD had relatively high foaming ability (150.24 ± 5.34°C) among three drying methods. However, the stability of emulsion and rheological properties prepared by FD was superior to other samples. Differential scanning calorimeter (DSC) showed CBTMP made by HD had the relatively good thermal stability (Tp = 91.49 ± 0.19 °C), followed by VD and FD. Digestive properties reflected that heating treatment could significantly increase its degree of hydrolysis *in vitro*. To sum up, the research could provide experimental guidance and theoretical support for the preparation method and utilization of CBTMP.

## Graphical Abstract

Drying methods affect physicochemical and functional characteristics of *Clanis Bilineata Tingtauica Mell* protein. 
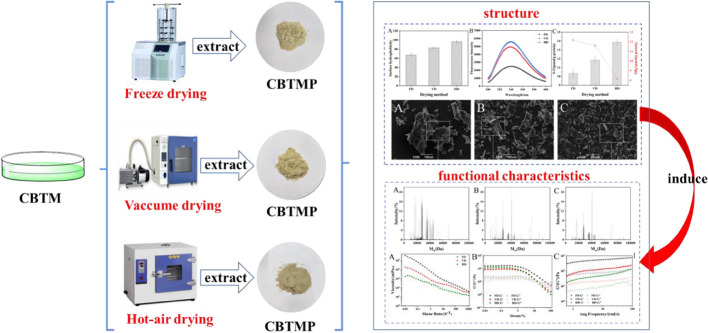

## Highlights

- Drying pretreatment significantly affected the structure and functional properties of CBTMP.
- CBTMP prepared by hot-air drying had the highest denaturation temperature but poor emulsifying stability.
- Freeze-dried protein was in a lower degree of oxidative denaturation than hot-air drying (and vacuum drying).
- The protein prepared by hot-air drying could be increased due to heating treatment.

## Introduction

With the continuous growth of the population and the deterioration of the ecology, traditional foods such as meat, seafood, and eggs had insufficient capacity to meet the ever-increasing demand of people (1). The development of new and sustainable insect protein food would become an inevitable trend in the future. Insect farming had also attracted more and more attention because of its low natural resource consumption, low carbon footprint, and short growth cycles (2). Moreover, insect protein was also a kind of natural full-price protein, which possessed highlighted nutritional characteristics and special functional properties, such as treating diseases and improving immunity (3). Additional, insect protein resources were readily available and they can be used as a kind of sustainable resource (3).

*Clanis Bilineata Tingtauica Mell* (CBTM) belonged to the *Lepidoptera Sphingidae*, *Cloudy Clanis Bilineata Tingtauica Mell* subfamily, *Clanis Bilineata Tingtauica Mell* species. As early as the Qing Dynasty, there are records of CBTM consumption. Compared with eggs and milk, CBTM not only had high protein content, but also the proportion of essential amino acids was reasonable. In addition, it had the function of lowering blood pressure and blood fat, having a good effect on human’s health (4, 5). At present, the study of insect resources was still weak and the study on CBTMP was rarely reported. Based on the circumstances, this research took the high content of CBTMP as the object to explore and make full use of its functional properties. Several studies suggested that processing condition including extraction methods and drying parameters, have an important effect on the structure (6). Due to their effects on protein structure, different drying methods can alter protein functional properties. The structure-function relationships of protein influence the way they interact with other ingredients of food (7, 8). Furthermore, protein powders were easier to store and transport than fresh or liquid forms of protein. Since drying is the critical step to make powders, the effects of CBTMP prepared by different drying methods were evaluated on its structure and functional characteristics. Freeze drying (FD), hot-air drying (HD), and vacuum drying (VD) were the three most commonly used drying techniques to convert protein into powder form. It was well known that freeze-drying could prevent the denaturation of most protein and minimize microbial reactions, but it was a kind of costly and time-consuming drying process (9). Vacuum drying was considered as a simple and popular technique, while it was also relatively expensive for large-scale production and it might destroy heat-sensitive substances (10). Hot-air drying was considered to be the simplest and most convenient technique, but it took a longer time, had lower energy utilization and easily led to the oxidative deterioration of materials (11). At present, it has been reported that different drying techniques had significant effects on the structure and functional properties of Antarctic krill protein (10), peanut protein (12) and so on. However, there are few studies on the effects of different drying methods on the protein functional properties of CBTMP. Because different kinds of proteins exhibited different sensitivity to drying-related stresses (12), it was of practical importance to explore the effect of drying methods on the structure and functional characteristics of CBTMP.

Hence, in this study the effect of these three drying methods on the surface hydrophobicity, Fourier Infrared Spectroscopy and surface morphology of CBTMP were investigated. Furthermore, functional characteristics including emulsifying properties, rheological properties, foaming properties, thermal stability and so on were also explored. This study hoped to provide experimental and theoretical support for the rational utilization of CBTMP.

## Materials and methods

### Materials

CBTM was purchased from Lianyungang City, Jiangsu Province. Other reagents were analytical grades. The soybean oil was bought from the local market. All solutions in the experiments were prepared by using ultrapure water (UP water) from a Millipore system (Millipore, Milford, MA, USA).

### *Clanis Bilineata Tingtauica Mell* protein extraction

The CBTM was dried, respectively, using a freeze-dryer (–40°C, 20 MPa), vacuum dryer (40°C, 0.1 MPa), and hot-air dryer (40°C). CBTM powders were defatted with petroleum ether at a flour ratio of 1:20 (w/v) for 12 h and 3 times prior to protein extraction. The defatted flour was placed in a fume hood for at least 24 h to evaporate residue petroleum ether. Defatted powder was used for protein extraction. In brief, the protein was extracted by alkaline dissolution and acid precipitation method according to our previous work (13). Finally, the pH of protein was adjusted to 7. All protein was dried by FD and was kept at-18°C for further analysis.

### Measurement of color and particle size

The color of CBTMP from different drying methods was measured using a Minolta Chroma Meter CR-300 colorimeter (Minolta Camera Co., Osaka, Japan). The color was collected, including L*(lightness), a* (greenness/redness) and b* (blueness/yellowness). Each protein sample was dispersed in UP water at a concentration of 0.2% (w/v). Particle size of protein was measured using a dynamic light scattering instrument (Zetasizer Nano-ZS, Malvern Instruments, Worcestershire, UK).

### Sodium dodecyl sulfate polyacrylamide gel electrophoresis

Sodium dodecyl sulfate polyacrylamide gel electrophoresis (SDS-PAGE) was conducted by a previous method (13, 14). The CBTMP powders were dispersed to UP water at a concentration of 0.2% (w/v) and mixed with an equal volume of SDS-PAGE sample buffer containing 5% β-mercaptoethanol. The solution was boiled for 2 min at 100 °C and centrifugated at 10,000 rpm for 5 min at room temperature. Then, the 10 μL mixtures was loaded on the gel slab which consisted of 12% resolving gel and 5% stacking gel. The voltages were set to 70 V and 110 V, respectively.

### Surface hydrophobicity and endogenous fluorescence spectra

The surface hydrophobicity (H_0_) of protein with the fluorescence probe 1-anilino-8-naphthalenesulfonate (ANS) (15) was determined with some modifications. First, CBTMP powders were added to UP water with concentrations of 0.02–0.1% (w/v). Then, 4 mL solutions were mixed with 60 μL of 8 mM ANS. The solutions were mixed for 30 S and incubated in the dark for 30 min. The fluorescent intensity of ANS–protein conjugate was conducted using excitation and emission wavelengths of 355 nm and 460 nm, respectively. The linear slope of the fluorescence intensity vs. concentration (mg/mL) was taken as the surface hydrophobicity. The excitation wavelength was 280 nm and the wavelength scanning ranged from 300 to 400 nm.

### Sulfhydryl/disulfide measurement

Free sulfhydryl groups were determined according to the literature method with slight modifications (16). CBTMP powders with a concentration of 0. 2% (w/v) were prepared by diluting with Tris-Gly buffer (pH 8.0). Then, 40 μL 5,5′-Dithiobis-(2-nitrobenzoic acid) (DTNB) (4 mg/mL) was added to 4 mL protein solution. The mixed solution was incubated in the dark for 30 min. The absorbance of sample was measured at 412 nm using an ultraviolet-visible (UV-VIS) spectrophotometer (UV 2600, Shimadzu, Japan). The content of SH was calculated using the following equation:


$${\text{C}}_{\text{SH}}=\frac{75.53^{*}\,{\text{D}}^{*}\,\text{A}}{\text{C}}$$

To measure S-S content, 2 mg/mL CBTMP solutions were prepared by 8 M urea Tris-Gly buffer. The β-mercaptoethanol (100 μL) was added to 4 mL CBTMP solution and kept for 30 min at 25 °C in the dark. Then, the mixtures were centrifuged and the precipitate was washed with 10 mL of 12% trichloroacetic acid (TCA) twice. The precipitate was dissolved in 15 mL of urea Tris-Gly buffer. The above solution was combined with 40 μL DTNB (4 mg/mL). The absorbance at 412 nm was measured after 30 min and the S-S content was calculated using the following equation:


$${\text{C}}_{\text{SS}}=\text{S}\,\frac{73.53^{*}\,{\text{D}}^{*}\,\text{A}}{\text{C}}-{\text{C}}_{\mathrm{SH})}^{*}\,0.5$$

In the above two equations, *D, C_*SS*_, A, C, C_*SH*_* were the dilution factor, disulfide bond content (μmol/g protein), the absorbance, the concentration of CBTMP and free sulfhydryl content (μmol/g protein), respectively.

### Water and oil holding capacity

The method of Ragab et al. (17) was used with minor modifications. One gram of protein powders was mixed with 10 ml of UP water (& medium-chain triglycerides) and then centrifuged at 10,000 rpm for 5 min. Afterward, the supernatant was decanted. The water holding capacity (WHC) and oil holding capacity (OHC) was expressed as the number of g of water or oil held by 1.0 g of protein sample.

### Foaming properties

Foaming characters were determined by the KJELDAHL’s method (18) with subtle modifications. Briefly, 0.2 g protein was dispersed into 20 mL UP water in a beaker. The initial volume was recorded as V_0_. The suspension was sheared for 1 min at 10,000 rpm with a high-speed homogenizer (T18, IKA, Germany). The total volume after shearing at 0 and 30 min was recorded as *V*_1_, *V*_2_, respectively. Foam capacity (FC) and foam stability (FS) was calculated as follows:

$$\text{FC}=\frac{{\text{V}}_{1}-{\text{V}}_{0}}{{\text{V}}_{0}}\times 100\%$$

$$\text{FS}=\frac{{\text{V}}_{1}}{{\text{V}}_{2}}\times 100\%$$

### Emulsifying properties

#### Emulsion ability index and emulsion stability index

Emulsion ability index (EAI) and emulsion stability index (ESI) were measured by a previous method (19) with subtle modifications. CBTMP powder was dispersed into UP water (10 mg/mL) and stirred until it was completely dissolved. The solutions were mixed with MCT (20%) and homogenized at 10,000 rpm for 3 min with a high-speed homogenizer (T18, IKA, Germany) to prepare emulsion. The particle size was measured by Mastersizer 2000 (Malvern Instruments Ltd., UK) and the optical microscope was used to observe the microscopic morphology of the emulsion. The emulsion diluted two times was placed on microslides and later covered with a coverslip. Then, 20 μL of emulsion was mixed well with 5 mL of 0.1% sodium dodecyl sulfate (SDS) solution. The absorbance of diluted emulsion was recorded at 500 nm and EAI (& ESI) was calculated using the following equations:


$$E\,A\,I=\frac{2^{*}\,T^{*}\,A_{0}^{*}\,D\,F}{C^{*}\,\varphi^{*}\,10000}$$

$$E\,S\,I=\frac{A_{0}}{A_{0}-A_{20}}\times t$$

Where T = 2.303; φ = 20%; t = 20 min; A_0_ and A_20_ were the absorbance at 0 and 20 min, respectively; DF, and C represented dilution factor, protein concentration (g/mL).

#### Rheological measurements

The rheological properties of emulsion were measured by Haake Rheometer with a parallel steel plate geometry (Φ = 35 mm). The following scan modes were measured according to our previous methods (20):

***The viscosity scan:*** the changes in viscosity were monitored with the shear rate from 0.01 to 1,000 S^–1^.

***The typical stress sweeps***: at a constant frequency of 1 Hz, the variation of storage modulus (G′) and loss modulus (G″) with strain (0.01–100%) was recorded.

***Frequency sweep tests***: in the linear viscoelastic region, the changes of G′ and loss G″ with frequency (0.01–100 Hz) were monitored. The phase angle (tan δ) was calculated as given by the equation:


$$\text{Tan}\delta =G"/\text{G}{}^{\prime }$$

### Measurement of thermal stability

Differential scanning calorimetry (DSC) was conducted as modified by Li et al. (21). Protein powders were accurately weighed and sealed in aluminum pans. The samples were heated from 30 to 150°C at a rate of 10°C/min with the nitrogen gas flow at 50 mL/min. The denaturation temperature and enthalpy were calculated using Universal Analysis 2000 software. A sealed empty DSC pan was used as the control.

### Digestive properties

#### Preparation of protein hydrolyzate

Enzymatic hydrolysis was determined according to the method of Brodkorb et al. (22) and Lu et al. (23). The CBTMP samples were hydrolyzed using pepsin and trypsin *in vitro*. All samples (2 mg/mL) were conducted at 37°C and the reactions were thermally terminated (boiling water bath, 10 min). At first, an enzyme-to-substrate (E/S) mass ratio of was 1:20 (w/w). For pepsin hydrolysis *in vitro*, the operation was carried out at pH 2 and sampled every 30 min. The pepsin hydrolysis reaction was performed for 2 h and pH was immediately adjusted to 8.5. Then, the operation was carried out at pH 8.5 *in vitro* and sampled every 30 min.

#### Degree of hydrolysis

The degree of hydrolysis (DH) was conducted according to the OPA method (24) with slight modifications. The 400 μL of samples were reacted with 3 mL OPA reagent accurately for 2 min, and the absorbance of 340 nm was determined. DH was calculated as follows:

$$\text{DH}\left(\%\right)=\text{h}/{\text{h}}_{\text{tot}}\times 100\%$$

$$\text{h}=0llows{\text{NH}}_{2}-\beta )/\alpha$$

Where β was 0.4 mequv/g; α equaled 1 mequv/g; h_*tot*_ was 8.8 mequv/g.

#### Digested products analysis

Peptide fractions were identified by a Q Exactive mass spectrometer (Thermo Scientific) and a Dionex Ultimate 3000 RSLCnano (Thermo Fisher Scientific) according to Li et al. (24) and Shen et al. (25). The peptide was dissolved in a gradient of solvent (80% acetonitrile, 0.1% formic acid) for 65 min at the flow rate 300 nL/min. The mass spectrometer ran in the data-dependent mode and automatically switched between MS and MS/MS. The separated peptides were directly entered into the mass spectrometer Thermo Scientific Q Exactive for on-line analysis, and the similarity of the peptides were analyzed by Venn diagram.

### Statistical analysis

The results were reported as mean ± standard deviation in these experiments. The figures were made with origin 2018 and the statistical analysis was performed with SPSS 21.0. *P* < 0.05 was considered to be statistically significant.

## Results and discussion

### Color, particle size, and sodium dodecyl sulfate polyacrylamide gel electrophoresis

**FIGURE 1:**
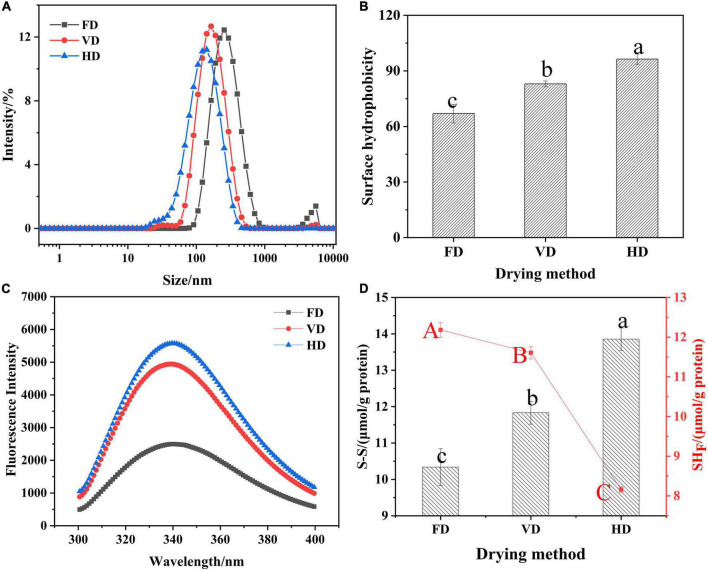
Effects of drying methods on particle size distribution **(A)**, surface hydrophobicity **(B)**, fluorescence spectroscopy **(C)** and the content of S-S (SH) **(D)**. Different shoulder letters indicate significant differences, and the same letters indicate no significant differences.

The color of sample was an important factor affecting its acceptance. Color characteristics of the CBTMP powders were significantly different (*p* < 0.05) (Table 1). Based on the color values (Supplementary Figure 1), the L* of VD (& FD) was higher than HD, indicating that the powders prepared by VD (& FD) was brighter. This may be due to the powder being exposed to oxygen during the HD process, which led to oxidative denaturation of the material (15). The results of particle size distribution were shown in Figure 1A.

**TABLE 1:** Effect of drying methods on color and the particle size of CBTMP.

Drying methods	Particle size/nm	PDI	D_4,3_/μm	Color		
				L*	a*	b*
**FD**	260.63 ± 2.69^c^	0.24 ± 0.0083	8.74 ± 0.01^a^	73.39 ± 0.40^b^	3.42 ± 0.15^b^	19.86 ± 0.26^c^
**VD**	149.10 ± 0.78^b^	0.21 ± 0.0124	9.68 ± 0.06^a^	74.41 ± 0.31^c^	2.87 ± 0.11^a^	18.01 ± 0.14^b^
**HD**	110.37 ± 1.02^a^	0.21 ± 0.0025	15.74 ± 0.05^b^	68.52 ± 0.66^a^	3.37 ± 0.16^b^	16.19 ± 0.26*^a^*

In the same column of data in the table, different shoulder letters indicate significant differences, and the same letters indicate no significant differences.

**FIGURE 2:**
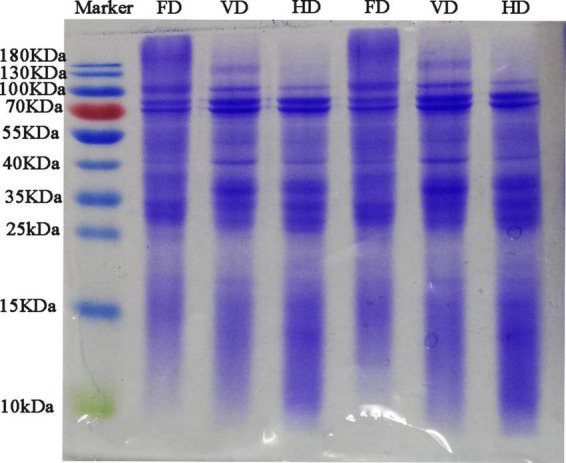
SDS-PAGE electrophoretic profiles of CBTMP with different drying methods.

The particle size presented bimodal distribution. It was obvious that the droplet size distribution of FD gradually moved toward the bigger diameters. The protein prepared by FD had the bigger mean particle size (260.63 ± 2.69 nm) compare with VD (149.10 ± 0.78 nm) and HD proteins (110.37 ± 1.02 nm) which was also exhibited in Table 1. Au et al. found that freeze-dried treatment can increase the particle size of yolk particles (26). The PDI value of CBTMP under different drying methods was less than 0.3, indicating that the solution was stable. The results showed that drying methods may obviously affect the colors and mean particle sizes. Figure 2 revealed that CBTMP was mainly distributed in the high molecular weight (30–130 KDa). It was clear that more intensive bands were presented for FD, which was consistent with the results of the research on quinoa protein isolate reported by Shen et al. (27). However, much weaker bands were observed for HD, and band intensity for VD was in between. FD was the mildest drying method owing to the lowest drying temperature among the three drying technologies (27). The drying temperature of HD was high and HD exposed to oxygen, which led to severe protein denaturation, aggregation and crosslinking, thus the bands were weaker.

### Surface hydrophobicity and endogenous fluorescence spectra

Surface hydrophobicity (H_0_) reflected the content of hydrophobic groups, determined emulsification and foaming properties and evaluated protein structure changes (15). As shown in Figure 1B, different drying methods had significant impact on surface hydrophobicity (*p* < 0.05). The surface hydrophobicity of CBTMP under different drying techniques was found to be in the following order: FD < VD < HD. The differences in surface hydrophobicity could be due to degree of oxidation of the protein. It was the heating treatment in the process of VD and HD that induced protein oxidation, which exposed hydrophobic groups leading to changes in the spatial structure of CBTMP. These results were in consistent with previous studies (28). In Figure 1C, it was clear that the emission fluorescence of protein solutions were observed at 340 nm mainly owing to tryptophan. In comparison with FD, the VD and HD curve presented a slight blue shift in the maximum emission wavelength and an increase of the fluorescence intensity, which implied more tryptophan residues was exposed (19). Notably, the fluorescence intensity of HD was significantly higher than that of VD (& FD), suggesting heating treatment might lead to the increase of fluorescence intensity (29).

### Analysis of free sulfhydryl group and disulfide bonds

The content of free sulfhydryl group (SH) as well as disulfifide bond (SS) of protein was exhibited in Figure 1D. The content reflected the degree of protein denaturation. Thus, the free sulfhydryl content of HD was the lowest and disulfide bond was the highest, implying the most intensive oxidation of free sulfhydryl group to form disulfifide bonds (30) during hot-air drying process. The results were in accordance with the changes in surface hydrophobicity (Figure 1B), which also indicated that a higher number of hydrophobic groups could increase the opportunity for adjacent SH groups to form SS groups. Lin et al. (10) also found that hot-air samples had the lowest content of SH. These differences were due to the longer drying time of HD than VD and therefore protein is affected by the prolonged heating. On the other hand, since HD did not remove oxygen, the samples were more susceptible to oxidation when exposed to oxygen.

### Water and oil holding capacities

**FIGURE 3:**
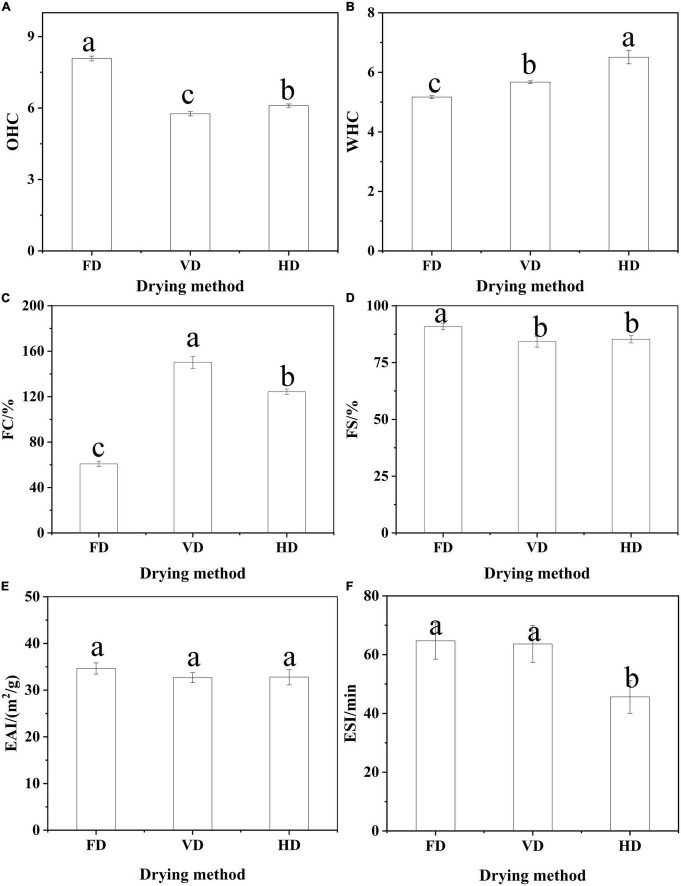
The effect of drying methods on WHC **(A)**, OHC **(B)**, foaming **(C,D)** and emulsifying properties **(E,F).** Different shoulder letters indicate significant differences, and the same letters indicate no significant differences.

The water (oil) absorption capacity was described as the ability of the protein to hold and retain an amount of added water (oil). In Figure 3A, the freeze-dried protein had significantly higher OHC than other proteins. Gong et al. (12) also reported the freeze-dried PPI had excellent OHC. However, relatively higher WHC was observed in the HD (Figure 3B). As these changes of protein conformation with increasing binding sites under high temperature could bring about higher WHC. The founding was in agreement with quinoa protein isolate (31), but different from protein isolate made from Australian chia seeds (15). The reason may be that the water and OHC of protein was affected by various factors, such as, structures, protein sources and other constituents in the materials.

### Foaming characteristics

Drying technology had a significant impact on the foaming characteristics of CBTMP (Figures 3C,D). The highest FC was observed for VD (150.24% ± 5.34), followed by HD (124.34% ± 2.48) and FD (60.83% ± 2.31). The FD samples with the biggest particle size could be the most slowly absorbed during stirring to generate more foam (27). Aluko and Monu (32) found enzymatically hydrolysis significantly improved foaming characteristics mainly owing to reduce particle size and form more stable interfacial membranes. Additionally, heating pretreatment also had a positive effect on foaming properties and the partial unfolding of protein was beneficial to improve foaming ability (33).

### Emulsifying properties

#### Emulsion ability index and emulsion stability index

**FIGURE 4:**
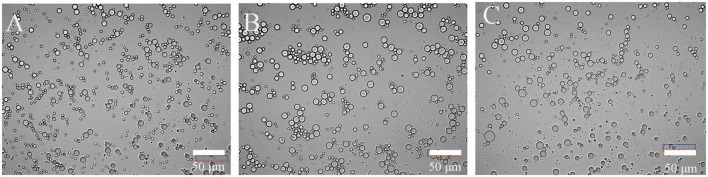
Optical microscope of emulsion prepared by CBTMP **(A)** -FD, **(B)** -VD, and **(C)** -HD).

Emulsifying capacity index (EAI) and emulsifying stability index (ESI) played an indispensable role in the food industry. The results of EAI and ESI were shown in Figures 3E,F. Different drying methods had no significant effect on its EAI (*p* > 0.05). Similar research was also reported that roasting did not significantly change the EAI of peanut protein concentrate (34). The protein of FD (& VD) had relatively higher ESI than HD. The better emulsifying stability of FD (& VD) may be attributed to better OHC and favorable dissociation at oil/water interface (35). Shen et al. and Feyzi et al. found that freeze dried protein had higher ESI (27, 28). In general, ESI depended on the particle size and rheological properties of the emulsion, the smaller the particle size was and the better the stability of the emulsion was. It can be seen from the d(4,3) (Table 1) and optical microscope (Figure 4) of the emulsion that the emulsification stability prepared by HD was poor.

#### Rheological characteristics

**FIGURE 5:**
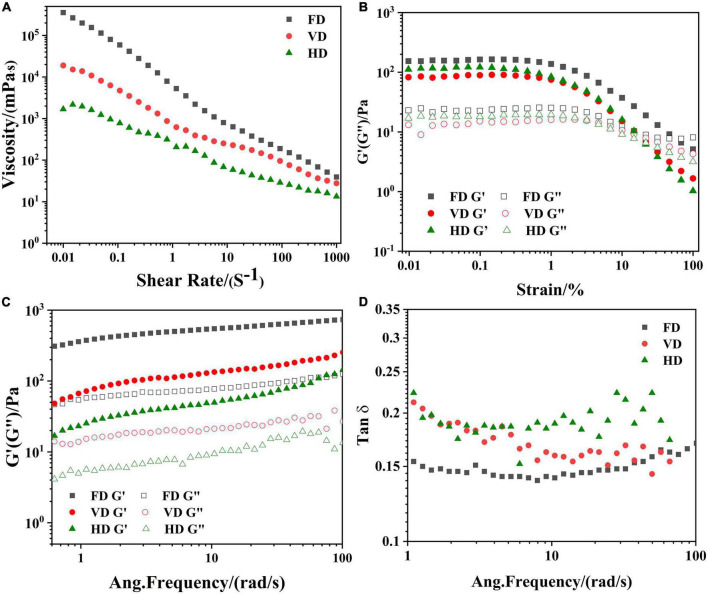
Influence of drying methods on the apparent viscosity 6 **(A)**, the variation of typical stress sweep 6 **(B)**; storage (G′, solid symbol), loss (G″, open symbol) change with frequency 6 **(C)**; phase angle 6 **(D)** of emulsion stabilized by CBTMP particles.

It was clear that rheological behaviors of emulsion were displayed in Figure 5. For viscosity scans (Figure 5A), all emulsion exhibited shear-thinning behaviors, which implied the emulsion were Non-newtonian fluid (36). In the variation of typical stress sweep (Figure 5B), as the shear stress increased, G′ and G″ remained basically unchanged, and then suddenly dropped, which indicated that the network structure of the emulsion was destroyed by the shear stress. Therefore, 1% was regarded as the linearity strain of the emulsion in the frequency scan. Besides, G′ was greater than G″, implying that the emulsion was mainly elastic (37). In Figure 5C, as the frequency increased, both G″ and G″ showed an increasing trend. Similar results were found in other proteins (27). Moreover, G′ was higher than loss G″ in all emulsion, further revealing emulsion was dominated by elastic structure. It was also observed the HD exhibited much lower G′ and G″, indicating the formation of weaker structure (38). On the contrary, the FD displayed the highest G′ and G″, implying the strongest emulsion formed with the best resistance to stress induced rupture among the three drying methods. During freeze-drying, most of the intrinsic properties were retained which favored the forming of emulsion networks (39). What’s more, phase angle (Figure 5D) in all emulsion was less than 1, which further implied that the emulsion was mainly elastic.

### Thermal properties analysis

**FIGURE 6:**
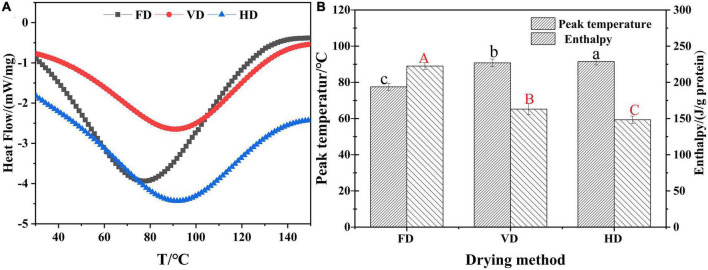
Effect of drying methods on thermodynamic characteristic of CBTMP. **(A)** Heat flow-T. **(B)** Peak temperature-drying method. Different shoulder letters indicate significant differences, and the same letters indicate no significant differences.

Differential scanning calorimetry (DSC) was used to determine the denaturation (& peak) temperature correlated with the thermal stability as well as the enthalpy reflecting the denaturation extent of proteins (40). In DSC profiles (Figure 6A), a major endothermic peak was observed for all samples and this was owing to protein denaturation during the drying process. HD of CBTMP had the highest peak temperature (91.49 ± 0.19°C) (Figure 6B), followed by VD (90.78 ± 0.21°C) and FD (77.53 ± 0.19°C). Enthalpy was usually used to reflect changes in protein structure. The value of enthalpy was much higher in FD sample (222.53 ± 0.49 J/g) compared to VD (163.05 ± 0.75 J/g) and HD (148.34 ± 0.50 J/g). The decrease of enthalpy reflected that less energy was required to unfolding of protein due to destruction of intramolecular bonds. These results revealed that drying methods could significantly influence thermal stability.

### Digestive properties

#### Degree of hydrolysis

**FIGURE 7:**
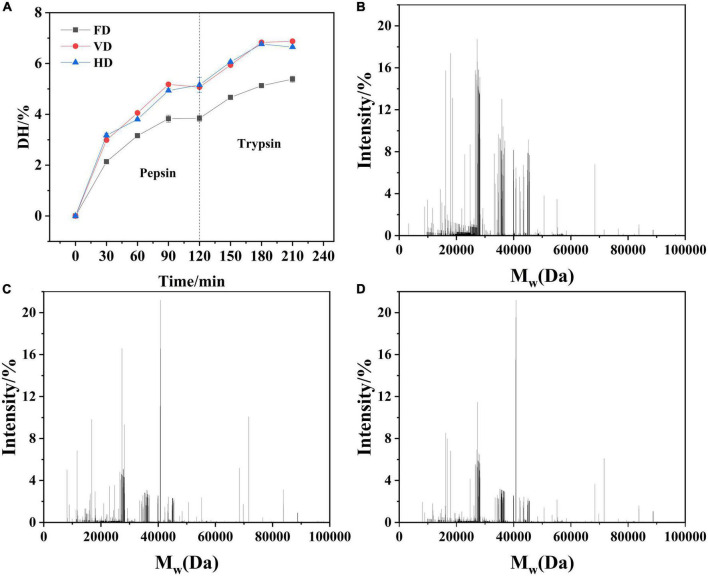
The degree of hydrolysis **(A)** and mass fingerprint (FD, **B**, VD, **C,** and HD, **D**) of digested products of CBTMP prepared by different drying methods.

The degree of hydrolysis (DH) was represented in Figure 7A. During vitro pepsin digestion, the degree of hydrolysis increased rapidly in the first 90 min, and then slowly increased. After 120 min, the degree of hydrolysis of FD, VD and HD reached 3.84, 5.07 and 5.16%, respectively. In trypsin digestion, the degree of hydrolysis increased sharply within the first 30 min, possibly because trypsin, as an endopeptidase, can cleave peptide bonds in the middle of the amino acid chain along the primary structure of its substrate. Therefore, the addition of trypsin could greatly increase DH and produce a large number of small molecule peptides. Subsequently, the degree of hydrolysis was stabilized, which may be due to the decreasing number of C-terminal peptides that can cleave lysine and arginine residues by trypsin and the activity of the enzyme in the reaction system was lowered. The degree of hydrolysis presented FD was the lowest. Studies (41) have reported that heating treatment could obviously increase the degree of hydrolysis, which was consistent with our results. Additionally, from the perspective of enzyme, the binding site of pepsin (trypsin) and protein was in the area of the hydrophobic group (42). Thus, the surface of FD had fewer hydrophobic groups and fewer binding site, the degree of hydrolysis was the lowest among three drying methods.

#### Digestion product analysis

After hydrolysis *in vitro*, the FD peptides’ molecular weight distribution (Figures 7B–D) was in the range of 20,000–30,000 Da. However, the peptides of VD and HD digestion products were mainly concentrated between 30,000 and 40,000 Da. We speculate that it may be due to the aggregation of proteins during the heating process, leading to an increase of molecular weight. Li et al. reported that as the degree of oxidation increased, the molecular weight of the peptide segment gradually increased after WMP digestion (24). Supplementary Figure 2 visually displayed the common peptides and unique peptides of CBTMP digestion products. There were 572 common peptides in the digested products of different drying treatments, which implied that these peptides were not affected by the drying methods. However, different drying methods had their own unique peptides, indicating that the digested products were significantly affected by the drying pretreatment. After FD treatment, the digested products of CBTMP identified 640 unique peptides. After VD pretreatment, the digested products identified 120 unique peptides. Smaller molecular weight oligopeptides or even amino acids may be produced due to the higher degree of hydrolysis of VD, which was difficult to identify.

## Conclusion

In this study, CBTMP was prepared using freeze drying, vacuum drying and hot-air drying. Results exhibited the protein of FD was less denatured, had better functional properties (ESI, OHC and rheological behavior) among three drying methods. This could be due to the fact that FD had a lower drying temperature and avoid the oxidation caused by the contact of the substance with oxygen. In addition, HD had the higher surface hydrophobicity and free sulfhydryl groups, which indicated that the degree of oxidative denaturation was obvious. However, HD possessed relatively high thermal stability compared with VD and FD. The CBTMP prepared by VD had the highest foaming and the degree of hydrolysis, which was mainly due to the fact that heating treatment obviously increases foaming properties and the degree of hydrolysis. In conclusion, drying methods had significant effects on structure, as well as on functional properties. Therefore, the study would have important significance for the development and rational utilization of CBTMP and benefit their utilization as a new protein material. However, the mechanism of the effect of drying methods on its functional characteristics needs further exploration.

## Data availability statement

The original contributions presented in this study are included in the article/Supplementary material, further inquiries can be directed to the corresponding author/s.

## Author contributions

SW: conceptualization, methodology, data curation, and writing—original draft. LN: data curation, and writing—review and editing. BZ: methodology, investigation, and data curation. YP: investigation, formal analysis, and writing—review and editing. XY: methodology and writing—review and editing. YS: project design and writing—review and editing. SL: project conceptualization, project administration, resources, writing—original draft and review, and editing. All authors contributed to the article and approved the submitted version.
