# Supplementary material for: Drying methods affect physicochemical and functional characteristics of Clanis Bilineata Tingtauica Mell protein
Source: Front Nutr. 2022 Nov 9;9:1053422. doi: 10.3389/fnut.2022.1053422 (PMC9682113; doi:10.3389/fnut.2022.1053422)

**Supplement material**

**Supplementary figure captions**

Figure S1 Protein powder appearance (A-FD, B-VD and C-HD)

Figure S2 Venn diagram of digested products of CBTMP with different drying method

**Figure S1**

**
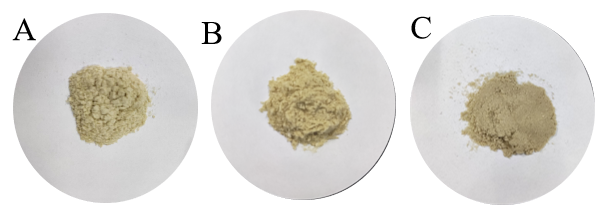
**

**Figure S2**


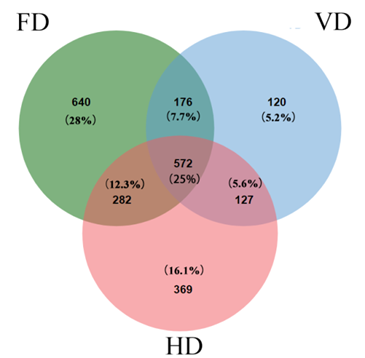

Supplement: Supplementary file 1 [file Data_Sheet_1.docx]
